# Comparison of radial and meander-like breast ultrasound with respect to diagnostic accuracy and examination time

**DOI:** 10.1007/s00404-020-05554-x

**Published:** 2020-05-03

**Authors:** Claudia Jäggi-Wickes, Pascale Brasier-Lutz, Sabine Schaedelin, Rosemarie Burian, Cora-Ann Schoenenberger, Rosanna Zanetti-Dällenbach

**Affiliations:** 1 Department of Obstetrics and Gynecology, University Hospital Basel, Spitalstrasse 21, 4056 Basel, Switzerland; 2 Department of Clinical Research, Statistics and Data Management, University Basel, Schanzenstrasse 55, 4031 Basel, Switzerland; 3 Department of Chemistry, University Basel, BioPark 1096, Mattenstrasse 24a, 4058 Basel, Switzerland; 4 Gynecology/Gynecologic Oncology, St.Claraspital Basel, Kleinriehenstrasse 30, 4002 Basel, Switzerland

**Keywords:** Diagnostic accuracy, Ductosonography, Examination time, False negative, Meander-like breast ultrasound, Radial breast ultrasound

## Abstract

**Purpose:**

To prospectively compare the diagnostic accuracy of radial breast ultrasound (r-US) to that of conventional meander-like breast ultrasound (m-US), patients of a consecutive, unselected, mixed collective were examined by both scanning methods.

**Methods:**

Out of 1948 dual examinations, 150 revealed suspicious lesions resulting in 168 biopsies taken from 148 patients. Histology confirmed breast cancers in 36 cases. Sensitivity, specificity, accuracy, PPV, and NPV were calculated for r-US and m-US. The examination times were recorded.

**Results:**

For m-US and r-US, sensitivity (both 88.9%), specificity (86.4% versus 89.4%), accuracy (86.9% versus 89.3%), PPV (64.0% versus 69.6%), NPV (both 98.3%), false-negative rate (both 5.6%), and rate of cancer missed by one method (both 5.6%) were similar. The mean examination time for r-US (14.8 min) was significantly (*p* < 0.01) shorter than for m-US (22.6 min).

**Conclusion:**

Because the diagnostic accuracy of r-US and m-US are comparable, r-US can be considered an alternative to m-US in routine breast US with the added benefit of a significantly shorter examination time.

## Introduction

In conventional breast ultrasound (US), the probe is typically moved in a meander-like manner in two orthogonal planes. Although this reveals the specific breast structures at different angles, it does not show them in their anatomical context, whereas radial breast ultrasound visualizes the entire extension of the anatomic structures of the breast, specifically ducts and lobules. Also, the nipple–areola complex, which often poses a diagnostic problem due to acoustic shadowing produced by the intricacy of the anatomic structures [1–3] can easily be delineated.

Radial breast ultrasound (r-US), also called ductosonography, was introduced by Rosensweig et al. [1]. It is mostly used in case of nipple discharge [4, 5] to examine dilated ducts and to visualize intraductal papillomas or other intraductal pathologies [6] in combination with conventional meander-like ultrasound (m-US). However, r-US is usually not applied on its own in routine clinical practice, mainly due to the width constraints of conventional probes (50 mm) which do not allow an efficient radial screening of the breast. Even after a wider probe (92 mm) became commercially available, the number of institutions or examiners performing r-US as routine breast ultrasound procedure remained limited although a number of authors and institutions consider r-US a viable alternative to m-US [3, 7, 8]. Consequently, only a handful of studies have been published where breast ultrasound was performed by radial and not by meander-like scanning [9–13]. However, to the best of our knowledge, the two scanning techniques have so far not been directly compared. In addition, diagnostic accuracy of r-US and the duration of the examination have not yet been comprehensively investigated. Therefore, we here compare the examination time and diagnostic accuracy between radial and meander-like scanning in the detection rate of malignant breast lesions, which were confirmed by histology.

## Materials and methods

Following approval by the local ethical committee (EKBB Nr. 123/11), we conducted this prospective single-center study from August 2011 to August 2014. The study included symptomatic women presenting with breast pain or palpable breast lumps, asymptomatic women with increased risk for breast cancer or women with dense breast tissue, and women with a history of breast cancer. Women younger than 18 years of age and women scheduled for minimal invasive breast biopsies were excluded from this study. As a result, the study comprised 1948 dual US examinations. All study participants signed the informed consent form in accordance with the World Medical Association Declaration of Helsinki.

Women of an unselected, consecutive, mixed collective were examined by conventional m-US and r-US on the same day by different examiners. Demographic data collection and physical breast examination were performed before the ultrasound examinations. All study subjects received a complete, bilateral r-US and m-US in random order, each of which was performed by independent examiners. Both examiners had full knowledge of the clinical findings, and where available, of mammographic results. However, the findings of the US examination were masked to the other examiner.

A designated research fellow specialized in gynecology and obstetrics but with limited prior experience in breast US performed all r-US. In addition to a yearly training in breast US that all examiners received, the research fellow received a theoretical and practical didactic training in r-US before study begin. M-US was performed by experts or, in the case of inexperienced examiners, under the supervision of an expert, as is common in teaching hospitals.

The r-US and m-US examinations were performed using two ultrasound machines of the same type (EUB-7500 V 16–53 Step 3.5, Hitachi; Hitachi Medical Systems Europe Holding AG, Zug, Switzerland). The m-US was performed with a 50 mm wideband, high-frequency (13–5 MHz) linear probe (EUP-L74M; Hitachi Medical Systems Europe Holding AG, Zug, Switzerland) and the r-US with a 92 mm wideband (10–5 MHz) linear probe (EUP-L53L; Hitachi Medical Systems Europe Holding AG, Zug, Switzerland) protected by a water-filled latex cover according to the manufacturer’s instructions (Hitachi Medical Systems Europe Holding AG, Zug, Switzerland). Each US examination was separately documented in the electronic patient records (ViewPoint^®^, Version 5; GE Healthcare GmbH, Munich, Germany).

To determine the duration of the examination, we recorded an image with a timestamp at the beginning and at the end of the ultrasound examination.

**Fig. 1:**
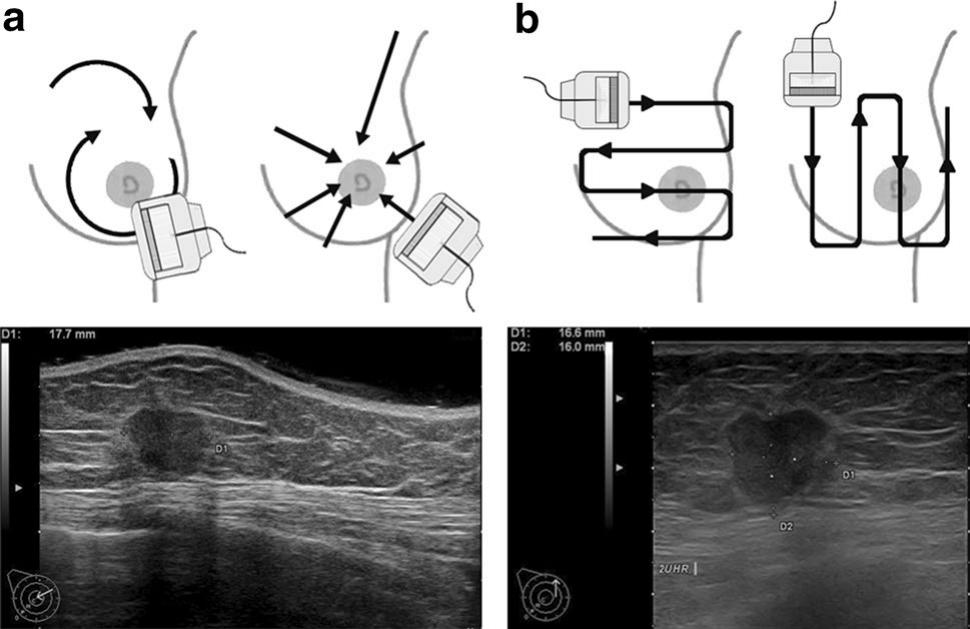
Radial and meander-like ultrasound. **a** Scheme of radial scanning movement (top) and corresponding ultrasound image (bottom). Left panel: radial movement and radial movement for the axillary tail; right panel: anti-radial movement. **b** Scheme of meander-like scanning movement in two orthogonal planes (top) and corresponding ultrasound image (bottom). Both ultrasound images are from the same 59-year-old patient diagnosed with invasive ductal breast cancer

Both breast US examinations were conducted with the woman lying in the same oblique supine position with her ipsilateral arm raised and her hand placed behind the head to flatten the breast tissue. R-US was performed as described by Teboul [14]. In brief, the probe was first moved clockwise around the nipple in a radial fashion (Fig. 1a, upper left panel). Then the upper outer quadrant of the breast was swept radial and anti-radial to explore the axillary tail. Finally, the probe was moved perpendicular to the ducts (anti-radial scan; Fig. 1a, upper right panel). For m-US, scanning was performed in two orthogonal planes with a meander-like probe movement (Fig. 1b) for each plane. Scanning of the axilla was routinely included. Representative ultrasound images for each method are shown in the bottom panels of Fig. 1.

We determined the dimensions of each sonographic lesion based on recordings in two orthogonal directions. Size, localization, morphologic characteristics of the lesion, and their BI-RADS classification according to the BI-RADS Atlas [15] were documented in the electronic patient records.

Breast lesions characterized as BI-RADS 4 or 5 and as BI-RADS 3 in case of an increased risk, were biopsied (sonographic-guided fine needle aspiration, core needle biopsy or vacuum-biopsy) and a cytologic or histopathologic diagnosis was obtained.

All data on patient and lesion characteristics extracted from the electronic patient records were entered into *R* (R Development Core Team 2018, Vienna, Austria) for data analysis.

## Statistical methods

Age, family history, and personal history were summarized in relation to the diagnosis. Family and personal histories are presented as frequencies and percentages. For the patient age, the mean and standard deviation were calculated and the minimum and maximum values were determined. The difference between benign and malignant lesions for different patient characteristics was compared using *t* and Chi-square tests. The length of the examination was calculated from the timestamp on the US image recorded at the beginning and at the end of the respective bilateral ultrasound examination. Examination time was compared between m-US and r-US using a Wilcoxon signed-rank test with continuity correction.

The results from the histology served as gold standard. Where lesions were missed by r-US or by m-US, the lesions were considered normal breast tissue and were interpreted accordingly for statistical analysis. For both methods, the sensitivity, specificity, and accuracy were calculated with 95% confidence intervals (CIs). The CIs were estimated according to Blaker [16]. *P* values were calculated using the exact McNemar’s test [17]. Positive and negative predictive values were calculated together with corresponding 95% CIs, and the respective *p* values were calculated [17]. The negative predictive value was calculated either including or excluding missed cancers. Only lesions described by both methods were compared for *p* value calculation.

The proportion of true positive, false negative, and cancers missed by one of the scan methods were calculated for malignant lesions. Correspondingly, the proportion of true negative and false positive, and the proportion of benign lesions not revealed were calculated for benign lesions. For data comparison between the two scan methods, an exact McNemar’s test was used.

Lesions from the same subject were considered independent. All analyses were performed by *R*. No correcting for multiple testing was performed.

## Results

We performed 2327 US examinations including both, r-US and m-US. We excluded 379 examinations (2 examinations in male patients, 18 examinations of patients younger than 18 years, 56 incomplete US examinations, 128 incomplete informed consent forms, and 175 incomplete datasets). Out of the remaining 1948 eligible dual US examinations, 150 (7.7%) ultrasound examinations of 148 patients revealed 168 suspicious lesions. Breast cancers were diagnosed in 36 lesions (1.8%). Because the remaining 1798 dual US examinations revealed no or unsuspicious lesions, no biopsy was performed.

Table 1 summarizes the general patient characteristics of the study population. Patients with no or no suspicious breast lesion had a mean age of 48.7 years, while those with a suspicious breast lesion a mean age of 47.1 years. Of the latter, women diagnosed with breast cancer had a mean age of 57.8 years and were significantly (*p* < 0.001) older than women with a benign diagnosis who had a mean age of 44.1 years. The incidence of a positive personal history of breast cancer or positive family history in patients with suspicious lesions and patients with no or no suspicious lesions that did not require a breast biopsy was similar.

**Table 1:** Patient characteristics

	All study subjects			Patients with suspicious breast lesions (*n* = 148)		
	No or no suspicious breast lesions	Suspicious breast lesions	*p* value	Benign histology	Malignant histology	*p* value
Number of patients (%)	1251 (100)	148 (100)		115 (100)	33 (100)	
Mean age in years (min, max) [± SD]	48.7 (18, 88) [± 14.3]	47.1 (19, 86) [± 14.7]	0.229	44.1 (19, 86) [± 14.0]	57.8 (30, 79) [± 12.1]	< 0.01
Personal history of breast cancer (%)	47 (3.8)	3 (2.0)	0.402	2 (1.7)	1 (3.0)	0.520
Negative family history (%)	894 (71.5)	95 (64.2)	0.081	74 (64.3)	21 (63.6)	0.485
Positive family history (%)	357 (28.5)	53 (35.8)		41 (35.7)	12 (36.4)	
Breast cancer	319	43		32	11	
Ovarian cancer	1	2		2	0	
Breast and ovarian cancer	14	2		1	1	
Endometrial cancer	10	6		6	0	
Breast and endometrial cancer	13	0		0	0	

All 168 suspicious lesions, 73 (43.5%) of which were clinically palpable, were clarified by sonographic guided fine needle aspiration (*n* = 10, 6.0%), core needle biopsy (*n* = 146, 86.9%), or vacuum biopsy (*n* = 12, 7.1%). The histopathologic/cytologic diagnoses revealed a malignancy in 21.4% (*n* = 36) and a benign histology in 78.6% (*n* = 132) of the lesions. The benign histologies comprised 6 (4.5%) B3 lesions, 50 (37.9%) fibroadenomas, 41 (31.1%) cases with fibrosis/sclerosis, and 35 (26.5%) other benign findings. All 36 carcinomas were diagnosed based on a core needle biopsy. Two (5.6%) ductal carcinoma in situ (DCIS), three (8.3%) invasive lobular carcinomas, and 31 (86.1%) invasive ductal carcinomas were identified. The final BI-RADS assessment based on m-US and r-US for all benign and malignant lesions and information whether or not the lesion was palpable or visible on mammographic images is presented in Table 2.

**Table 2:** Findings of clinical palpation and mammography and final BI-RADS assessment for benign and malignant breast lesions

	Malignant lesions				Benign lesions				
	Malignant lesions	Invasive ductal	Invasive lobular	DCIS	Benign lesions	B2 lesions	Fibrosis/sclerosis	Fibroadenoma	B3 lesions
	*n* (%)	*n* (%)	*n* (%)	*n* (%)	*n* (%)	*n* (%)	*n* (%)	*n* (%)	*n* (%)
	36 (100)	31 (86.1)	3 (8.3)	2 (5.6)	132 (100)	35 (26.5)	41 (31.1)	50 (37.9)	6 (4.5)
Meander-like ultrasound									
BI-RADS 5	15 (41.7)	12 (80.0)	3 (20.0)		1 (0.8)^+^		1 (100)		
BI-RADS 4	17 (47.1)	16 (94.1)		1 (5.9)	17 (12.9)^+^	5 (29.4)	7 (41.2)	4 (23.5)	1 (5.9)
BI-RADS 3	2 (5.6)*	1 (50.0)		1 (50.0)	104 (78.7)	29 (27.9)	30 (28.8)	41 (39.4)	4 (3.9)
BI-RADS 2	0 (0.0)				9 (6.8)	1 (11.1)	3 (33.3)	5 (55.6)	
Missing	2 (5.6)^҂^	2 (100)			1 (0.8)^‡^				1 (100)
Radial ultrasound									
BI-RADS 5	15 (41.7)	13 (86.7)	2 (13.3)		2 (1.5)^+^		2 (100)		
BI-RADS 4	17 (47.1)	15 (88.2)	1 (5.9)	1 (5.9)	12 (9.1)^+^	2 (16.7)	6 (49.9)	2 (16.7)	2 (16.7)
BI-RADS 3	2 (5.6)*	1 (50.0)		1 (50.0)	100 (75.7)	27 (27.0)	27 (27.0)	42 (42.0)	4 (4.0)
BI-RADS 2	0 (0.0)				15 (11.4)	6 (40.0)	5 (33.3)	4 (26.7)	
Missing	2 (5.6)^҂^	2 (100)			3 (2.3)^‡^		1 (33.3)	2 (66.7)	
Palpation									
Lesion palpable	23 (63.9)	21 (91.2)	1 (4.4)	1 (4.4)	50 (37.9)	16 (32.0)	9 (18.0)	24 (48.0)	1 (2.0)
Lesion not palpable	8 (22.2)	6 (75.0)	1 (12.5)	1 (12.5)	73 (55.3)	18 (24.7)	29 (39.7)	22 (30.1)	4 (5.5)
Missing	5 (13.9)	4 (80.0)	1 (20.0)		9 (6.8)	1 (11.1)	3 (33.3)	4 (44.5)	1 (11.1)
Mammography									
Lesion detected	30 (83.3)	25 (83.3)	3 (10.0)	2 (6.7)	39 (29.5)	12 (30.8)	10 (25.6)	13 (33.3)	4 (10.3)
Lesion not detected	5 (13.9)	5 (100)			24 (18.2)	11 (45.8)	10 (41.7)	3 (12.5)	
Not performed ≤ 40 years	0 (0.0)				40 (30.3)	7 (17.5)	6 (15.0)	26 (65.0)	1 (2.5)
Missing	1 (2.8)	1 (100)			29 (22.0)	5 (17.2)	15 (51.8)	8 (27.6)	1 (3.4)

*False negative, ^+^False positive, ^҂^Missed cancers, ^‡^Benign lesion not revealed

As indicated by the asterisk in Table 2, two of the 36 breast cancers were falsely characterized as BI-RADS 3 by r-US and m-US (one DCIS and one triple negative invasive ductal carcinoma). This corresponds to a false-negative rate of 5.6%.

Two other breast cancers were missed by m-US but correctly identified by r-US whereas two different breast cancers were missed by r-US but correctly identified by m-US (Table 3). None of the cancers missed by either of the scanning methods were palpable. This results in cancer miss rate of 5.6% for either method (Table 4). A sensitivity of 88.9% was calculated for r-US and for m-US (Table 4).

**Table 3:** False-negative lesions and lesions missed by either scanning method

Histology	Meander-like ultrasound	Radial ultrasound	Lesion palpable	Lesion detected by mammography
False negative lesions				
DCIS	BI-RADS 3	BI-RADS 3	No	Yes
Inv. ductal	BI-RADS 3	BI-RADS 3	Yes	Yes
Cancers which were missed by one method				
Inv. ductal	Missed	BI-RADS 4	No	Yes
Inv. ductal	Missed	BI-RADS 4	No	Yes
Inv. ductal	BI-RADS 4	Missed	No	No
Inv. ductal	BI-RADS 4	Missed	No	No
Benign lesions which were missed by one method				
B3-Lesion	Missed	BI-RADS 3	No	Yes
Fibrosis	BI-RADS 4	Missed	No	Yes
Fibroadenoma	BI-RADS 3	Missed	No	No mammography
Fibroadenoma	BI-RADS 3	Missed	No	No mammography

**Table 4:** Diagnostic accuracy of conventional meander-like ultrasound and radial ultrasound

	Meander-like ultrasound			Radial ultrasound			*p* value	CI
	*n*	%	CI	*n*	%	CI		
Malignant lesions	36	100		36	100			
Cancers identified	34	94.4	[81.4; 99.0]	34	94.4	[81.4; 99.0]	1	[0.1; 13.8]
Cancers missed	2	5.6	[1.0; 18.6]	2	5.6	[1.0; 18.6]	1	[0.1; 13.8]
True positive	32	88.9	[74.2; 96.1]	32	88.9	[74.2; 96.1]	1	[0.1; 13.8]
False negative^a^ (BI-RADS 3)	2	5.6	[1.0; 18.6]	2	5.6	[1.0; 18.6]	1	[0.0; 13.8]
False negative^b^ (BI-RADS 3 and missed cancers^c^)	4	11.1	[3.9; 25.8]	4	11.1	[3.9; 25.8]	1	[0.1; 13.8]
Benign lesions	132	100		132	100			
Benign lesions identified	131	99.2	[96.1; 100.0]	129	97.7	[93.7; 99.4]	0.63	[0.0; 4.2]
Benign lesions missed	1	0.8	[0.0; 3.9]	3	2.3	[0.6; 6.3]	0.63	[0.0; 4.2]
True negative	113	85.6	[78.6; 90.8]	115	87.1	[80.5; 92.1]	0.79	[0.2; 2.5]
False positive^d^ (BI-RADS 4 or 5)	18	13.6	[8.4; 20.6]	14	10.6	[6.2; 16.8]	0.39	[0.5; 9.1]
False positive^d^ (BI-RADS 4 or 5 and missed lesions^e^)	19	14.4	[9.2; 21.4]	17	12.9	[7.9; 19.5]	0.79	[0.2; 2.5]
Diagnostic accuracy								
Sensitivity		88.9	[74.2; 96.1]		88.9	[74.2; 96.1]	1	[0.1;13.8]
Specificity		86.4	[79.4; 91.6]		89.4	[83.2; 93.8]	0.39	[0.1; 1.9]
Accuracy		86.9	[81.1; 91.6]		89.3	[83.8; 93.4]	0.45	[0.2; 1.8]
PPV^f^		64.0	[50.0; 76.4]		69.6	[54.4; 82.2]	0.90	[0.25; 3.38]
NPV^g^ (excluding missed cancers)		98.3	[94.2; 99.7]		98.3	[94.4; 99.7]	0.45	[1.0; 1.0]
NPV^g^ (including missed cancers)		96.6	[91.8; 98.8]		96.7	[92.1; 98.9]	0.95	[0.97; 1.03]

^a^The same two breast lesions were classified as BI-RADS 3 by both methods

^b^False negative and NPV were calculated excluding and including missed cancers and ^d^false positive was calculated excluding and including missed lesions^e^. In general, studies do not include a second US examination and thus, the cancer miss rate and the number of missed benign lesions remain unknown. Consequently, missed cancers/missed benign lesions are not represented in the calculation of false negative/false positive values and NPV

^c^Cancers missed by one method but correctly identified by the other

^e^Benign lesions not revealed by one of the two methods

^f^PPV, positive predictive value; ^g^NPV, negative predictive value

The false positive rate was 13.6% (*n* = 18) for m-US and 10.6% (*n* = 14) for r-US with a specificity of 86.4 and 89.4%, respectively (Table 4). Among the benign lesions, ten were falsely classified as BI-RADS four or BI-RADS five by both methods. Additionally, m-US classified 8, and r-US 4 benign lesions as BI-RADS 4 or 5, while they were correctly identified as benign by the other method. The number of benign lesions not revealed were one (0.8%) for m-US and three (2.3%) for r-US. Benign lesions which were missed by either of the scanning methods are listed in Table 3.

The diagnostic accuracy is similar for r-US and m-US. To evaluate whether one of the methods offers potential patient benefits, we compared the average time needed for a bilateral whole breast examination (Table 5).

**Table 5:** Duration of bilateral meander-like ultrasound versus radial ultrasound examination

	Meander-like ultrasound	Radial ultrasound	*p* value
Mean examination duration revealing suspicious lesions requiring breast biopsy (min)	22.6	14.8	< 0.01
(min, max) [± SD]	(4.7, 74.0) [± 12.8]	(3.9, 47.0) [± 7.6]	
Malignant lesions	26.6	14.8	< 0.01
(min, max) [± SD]	(4.7, 69.0) [± 18.0]	(3.9, 38.5) [± 7.1]	
Benign lesions	21.7	14.8	< 0.01
(min, max) [± SD]	(7.1, 74.0) [± 11.2]	(5.2, 47.0) [± 7.8]	
Mean examination duration revealing no or no suspicious lesions (min)	13.3	7.6	< 0.01
(min, max) [± SD]	(2.0, 58.2) [± 6.2]	(2.3, 64.2) [± 5.8]	
No lesion detected	9.7	4.0	< 0.01
(min, max) [± SD]	(4.2, 22.2) [± 3.8]	(2.3, 9.5) [± 1.3]	
1 lesion detected	13.2	6.6	< 0.01
(min, max) [± SD]	(4.7, 32.3) [± 5.9]	(2.4, 24.5) [± 3.3]	
> 1 lesion detected	14.6	9.3	< 0.01
(min, max) [± SD]	(2.0, 58.2) [± 6.6]	(2.7, 64.2) [± 6.8]	
Mean examination duration of all examinations (min)	15.0	8.9	< 0.01
(min, max) [± SD]	(2.0, 74.0) [± 8.6]	(2.3, 64.2) [± 6.7]	

The overall duration of examinations revealing suspicious lesions was 22.6 min for m-US while a significantly shorter length of 14.8 min was found for r-US (*p* < 0.01). As shown in Table 5, the trend of a faster examination by r-US was observed independent of lesion histology. Moreover, the overall examination time in study patients whose US did not reveal a suspicious lesion was 13.3 min for m-US and 7.6 min. In addition, comparison of the examination time and diagnostic performance for the first versus the last 50 r-US of suspicious lesions revealed that the r-US examiner became more versed with regard to examination time at no loss of diagnostic accuracy (Table 6).

**Table 6:** Comparison of the first and last 50 radial ultrasound examinations revealing suspicious lesions

	First 50 radial ultrasound examinations	Last 50 radial ultrasound examinations	*p* value
Duration of radial ultrasound examination			
Mean time (min)	19.8	9.9	< 0.01
(min, max) [± SD]	(5.2, 47.0) [± 8.6]	(3.9, 17.6) [± 3.4]	
Diagnostic accuracy			
Sensitivity [CI]	70.0 [38.1; 91.3]	91.7 [63.4; 99.6]	0.45
Specificity [CI]	90.0 [76.8; 96.5]	86.8 [72.8;94.7]	1
Accuracy [CI]	86.0 [73.4; 93.7]	88.0 [76.4; 94.6]	1

These data corroborate that the r-US is significantly shorter than m-US, independent of whether or not a lesion was detected.

## Discussion

To the best of our knowledge, this study represents the first direct comparison of m-US and r-US. While the diagnostic accuracy of r-US and m-US regarding the detection of breast lesions were comparable, we found that the average examination time for a whole bilateral breast ultrasound was significantly shorter for r-US than for m-US for all patients independent of whether or not a suspicious lesion was detected.

In our study, 36 breast cancers were diagnosed out of 1948 combined US examinations. This results in a cancer detection rate of 1.8%. Under similar conditions, i.e. an unselected mixed study collective, cancer detection rates ranging from 0.83 [18] to 3.2% [19] have been reported. For m-US, we found a sensitivity of 88.9% and a specificity of 86.4% which is within the range of previously reported values [18–21]. A recent meta-analysis [22] published a sensitivity of 87% and specificity of 72%. It is noteworthy that for r-US, we observed an equally high sensitivity, while the specificity was slightly higher than for m-US (89.4% versus 86.4%).

The diagnostic accuracy of r-US was 89.3% which corresponds to the highest values found for m-US [19, 20]. Similarly, PPV (69.6%) and NPV (98.3%) for r-US were in the range compared to published m-US data [20, 21, 23–25].

The only values published for r-US that we are aware of represent combined data from r-US and mammography [9]. Nevertheless, sensitivity (90.3%), specificity (78.5%), and diagnostic accuracy (81.8%) compare reasonably well to our data (88.9, 89.4, and 89.3%, respectively). The false-negative rate of r-US in combination with mammography (9.7%) was higher than what we found for r-US alone (5.6%). Although the false-negative rates for m-US vary in the literature (3.6–7.5%) [20, 25, 26], we found the same value for r-US and m-US (5.6%). Two lesions were wrongly classified as BI-RADS 3 lesions by m-US and r-US, while histology revealed a DCIS and an invasive ductal triple negative breast cancer. It is conceivable that triple negative breast cancers may be wrongly classified because their sonographic appearance often lacks malignant features [27].

Two malignancies were not detected by m-US and two others were not detected by r-US (Table 3). However, each of them was identified by the other method which results in a cancer miss rate of 5.6% for either method. One of the carcinomas missed by r-US was localized in the upper outer quadrant close to the axilla. This emphasizes the importance to also sweep the outer upper quadrant with the radial probe. The number of benign lesions not revealed by one, but by the other method was one (0.8%) for m-US, and three (2.3%) for r-US. Overall, in our study 98.2% of all lesions were detected by m-US and 97.0% by r-US. In comparison, Kim et al. reported a detection rate of 93.9% for m-US [28]. According to Berg et al. [29], individual examiners detected between 49 and 66% of the lesions by m-US. In a breast phantom study [30], a median of 14 out of 17 lesions (82%) were detected. The detection rate of 97.0% for r-US is similar to our detection rate for m-US and higher than the published rates for m-US.

The mean examination time for a bilateral whole breast m-US was 13.3 min for patients without or with unsuspicious breast lesions, and 22.6 min for patients with suspicious breast lesions. Mean examination times of 20.8 min in a mixed collective (ranging from 2–90 min) [31] and 31 min in patients with three and more lesions (ranging from 3–59 min) [29] have been reported for bilateral m-US. In contrast, the present study reveals a significantly shorter examination time for r-US, which might be related to the increased width of the probe. With a mean examination time of 7.6 min for patients without or with unsuspicious lesions, and 14.8 min for patients with suspicious breast lesions. Our data show good agreement with Rosensweig [1] who reported an examination time of 14 min for r-US. Some examiners might perform meander-like or radial ultrasound only in one plane, which would evidently shorten the examination time. However, in this study, the reported examination time refers to two-plane scanning for m-US and r-US. Thus, we can conclude that in general, radial sonographic breast examination takes less time independent of the histology or the number of breast lesions.

Based on the study design, m-US and r-US could not be carried out by the same examiner, because knowledge of the first US results would bias the second examination, which constitutes a limitation of our study. M-US was performed by experts or supervised inexperienced examiners as it is common in teaching hospitals. Therefore, we cannot exclude that when examiners with less experience performed m-US examinations, times were slightly increased compared to experienced examiners. In addition, not all patients agreed to participate in the study and thus, the study collective may not fully represent the consecutive, mixed population of an outpatient breast clinic. Out of the 1948 dual m-US/r-US examinations, 150 examinations revealed 168 suspicious lesions. Unsuspicious lesions (BI-RADS 3 lesions with no additional risk factors, and BI-RADS 2 lesions) were not biopsied. The absence of proven negative results might be considered a limitation. However, our data reflect examination procedures common to teaching hospitals and routine clinical settings.

Radial ultrasound is a viable alternative to conventional meander-like ultrasound in routine breast examination. Sensitivity, specificity, and false-negative rate of the two methods are comparable. In addition, patients benefit from a significantly shorter examination time.
